# Excision of the External Carotid Artery in Cases of Inoperable Malignant Diseases of the Face

**Published:** 1900-12

**Authors:** William Perrin Nicolson

**Affiliations:** Atlanta, Ga.


### EXCISION OF THE EXTERNAL CAROTID ARTERY IN CASES OF INOPERABLE MALIGNANT DISEASES OF THE FACE.*

By WILLIAM PERRIN NICOLSON, M.D.,

ATLANTA, GA.

The first case was a sarcoma of the nose which began apparently as a polypus about eight months before. This was removed several times but recurred promptly after each removal. When seen a few weeks before the operation this had extended sufficiently to completely obstruct the nose and cause great pain by constant pressure. At the time of the operation this had progressed in a few weeks only, so that the growth pressing under the orbit had forced the right eye out of position and there had been also an extension upon the forehead upon the left side. The patient suffered intense pain, which required constant use of morphine for its relief. The right common carotid was excised on October 3d and the wound healed promptly. The enlargement upon the left side of the forehead broke down and the large abscess was opened

---

Read before the meeting of the Southern Surgical and Gynecological Association at Atlanta, Ga.

a few days after the operation. The pus from this, at the discharge from the nose, set up a violent ophthalmia from which the patient suffered for a week or ten days. Two weeks from the day of the first operation the carotid upon the left side was removed and very soon the symptoms improved in every respect, the patient was relieved of suffering, and the growth not only checked but it apparently began to recede, with the prospects of a material improvement in his condition.

The second case was one of inoperable sarcoma of the upper jaw of three months' duration and of very rapid growth. In this case the interval between the operations was longer than in the first, on account of the occurrence of a severe secondary hemorrhage on the seventh day, which was due to tying the vessel too close to the bifurcation. This first operation in this case relieved the patient of all the symptoms caused by the rapidly increasing pressure and the growth apparently subsided materially. The second operation had not been performed long enough to give much idea as to how much permanent decrease there would be in the tumor.

In commenting upon the operation it was claimed that in these cases the patient was simply doomed if nothing could be done, and this appeared to be the only recourse that offered any hope of benefit. He had performed various operations upon the external carotid artery in the cases of malignant disease, having tied the vessel twenty-six times, four of these being cases of double ligation. The operation had been not accompanied by any mortality. Little could be accomplished by simple ligation of even both carotida because the circulation was re-established so rapidly that the nutrition could not be cut off with any degree of permanence. The operation of excision as recommended by Dawbarn seemed to be the only procedure that offered any hope, and while this would not perhaps produce much permanent effect, yet it seemed undoubtedly true that the lives of patients could be much prolonged and their sufferings greatly lessened. The operation was one of considerable magnitude and dealt with structures of great importance anatomically, yet the result demonstrated that there was comparatively little danger in the performance of it.

